# Metal Surface Defect Detection Based on a Transformer with Multi-Scale Mask Feature Fusion

**DOI:** 10.3390/s23239381

**Published:** 2023-11-24

**Authors:** Lin Zhao, Yu Zheng, Tao Peng, Enrang Zheng

**Affiliations:** 1 School of Electrical and Control Engineering, Shaanxi University of Science and Technology, Xi’an 710021, China; 210612064@sust.edu.cn (L.Z.); 210611013@sust.edu.cn (T.P.); 2 School of Cyber Engineering, Xidian University, Xi’an 710126, China; yzheng@xidian.edu.cn

**Keywords:** defect detection, vision transformer, multi-scale feature fusion, pruning and merging

## Abstract

In the production process of metal industrial products, the deficiencies and limitations of existing technologies and working conditions can have adverse effects on the quality of the final products, making surface defect detection particularly crucial. However, collecting a sufficient number of samples of defective products can be challenging. Therefore, treating surface defect detection as a semi-supervised problem is appropriate. In this paper, we propose a method based on a Transformer with pruned and merged multi-scale masked feature fusion. This method learns the semantic context from normal samples. We incorporate the Vision Transformer (ViT) into a generative adversarial network to jointly learn the generation in the high-dimensional image space and the inference in the latent space. We use an encoder–decoder neural network with long skip connections to capture information between shallow and deep layers. During training and testing, we design block masks of different scales to obtain rich semantic context information. Additionally, we introduce token merging (ToMe) into the ViT to improve the training speed of the model without affecting the training results. In this paper, we focus on the problems of rust, scratches, and other defects on the metal surface. We conduct various experiments on five metal industrial product datasets and the MVTec AD dataset to demonstrate the superiority of our method.

## 1. Introduction

Surface defect detection on metal surfaces is widely used in various fields such as transportation [1,2,3], aerospace [4,5,6,7], and industrial manufacturing [8,9,10] in industrial production. Common defects that can be found on metal surfaces include deformation, rust, scratches, and others, which not only affect the aesthetics but also pose safety hazards. Therefore, surface defect detection plays an important role in production. However, there are major challenges in surface defect detection for products. Firstly, the number of defective products is relatively low compared to normal products, making it difficult to collect a sufficient number of defect samples. Secondly, appearance defects of the products are complex and diverse, and the morphological characteristics may vary greatly among different categories of defects. It is difficult to consider all defective products as a single effective category.

The most traditional detection method is manual visual inspection, but the results can be easily influenced by subjective factors. With the rise of machine vision technology, traditional machine vision detection mostly relies on image processing [11,12] and setting multiple thresholds for various types of defects in the images, which greatly increase the detection time and economic costs and have certain limitations. The performance of industrial surface defect detection based on deep learning techniques is continuously improved with an increase in sample data. It can directly extract defect features and overcome the limitations of traditional machine vision detection methods. Among them, fully supervised anomaly detection methods require labeled datasets. However, in practical applications, there exist various types of anomalies and the number of defect samples is limited, which lead to overfitting during the learning process and affect accuracy. In contrast, semi-supervised anomaly detection methods that require only a small amount of labeled data are widely applied.

Under the framework of semi-supervised learning, research has shown that methods based on image reconstruction can effectively address the challenges of limited samples and complex and diverse defects. Among them, methods based on generative adversarial networks (GANs) [13] and Autoencoders (AEs) [14] have made rapid developments in defect detection. Youkachen et al. [15] used a Convolutional Autoencoder (CAE) to reconstruct images and perform surface defect segmentation on hot-rolled steel strips. However, the low reconstruction accuracy led to a low defect detection precision. The f-AnoGAN proposed by Schlegl et al. [16] addresses the time-consuming issue of iterative optimization during training by introducing an encoder. Akcay et al. proposed the GANomaly [17] network, which encodes and decodes input images to reconstruct them and defines anomaly scores by encoding the latent vectors of input and reconstructed images. To enhance the reconstruction capability, Skip-GANomaly [18] uses an encoder–decoder convolutional neural network with skip connections to capture multi-scale features of the data distribution. However, emphasizing the differences between reconstructed and input images in anomaly scores resulted in inaccurate detection. Xudong Yan et al. proposed a Semantic Context-based Anomaly Detection Network (SCADN) [19], which used stripe masks of different widths and orientations to capture details and contextual information on different scales. However, the complexity and slow speeds of testing individual error maps were not conducive to real-world applications. On the other hand, by applying Transformers to image classification tasks, models like Vision Transformer (ViT), can recognize more details in images. AnoViT [20] and VT-ADL [21], designed based on ViT, use Transformers as encoders to minimize the differences between input and reconstructed images. VT-ADL added a Gaussian mixture density network after the encoder’s output to locate anomalies. However, these two methods have low accuracies and have not been widely applied.

In contrast to image reconstruction, the Discriminative Training-based Reconstruction Embedding Model (DRÆM) [22] introduces additional texture images as noise overlaid on normal images to create texture anomalies. Guodong Wang et al. proposed the Student–Teacher Network [23], where a pre-trained model serves as the teacher network to extract knowledge into a single student network with the same architecture. Deng, H et al. introduced the Reverse Distillation method [24], where the student network does not directly receive the original images but takes the teacher model’s single-class embedding as an input and target. However, the training of the aforementioned models requires a large amount of data, and insufficient data can lead to a decrease in model performance. Defard et al. designed a Patch Distribution Modeling (PaDiM) [25] framework, which uses a pre-trained CNN to generate feature and model normality by applying a multivariate Gaussian distribution to each position. Roth et al. followed the idea of the SPADE model [26] and proposed the PatchCore method for anomaly detection using patch-level features extracted from a pre-trained network on ImageNet. However, these methods rely on the accuracy of the feature extraction network, which can lead to feature learning biases.

**Figure 1:**
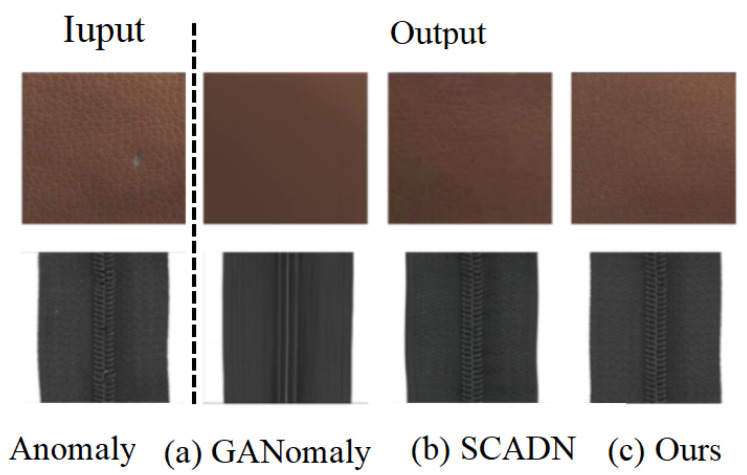
The visual comparison results of image reconstruction using GANomaly, SCADN, and our method show that, in comparison, their reconstructed images fail to accurately highlight the details of the original image. In contrast, our method achieves a restoration of the defective regions that is closer to the anomaly-free state, resulting in a more realistic representation of patterns and details.

In this paper, we propose a solution to address the issues mentioned above. To overcome the problems of a low accuracy, weak feature extraction capability, and poor reconstruction ability, we incorporate the ViT into a generative adversarial network to focus on capturing more details in images and achieve a Nash equilibrium between the generative and discriminative models. To address resource consumption, long training times, and low accuracies, we design complementary multi-scale block masks to obtain more semantic information while reducing the number of error maps and shortening the training time. As shown in Figure 1c, our method successfully detects anomalies in the input image and reconstructs an anomaly-free image.

In summary, this paper makes contributions in the following three aspects:

1. We propose a new network architecture that combines a ViT network with a generative adversarial network and introduces skip connections. The U-ViT [27] in the VIT-based architecture acts between the encoder and the decoder.
2. We design a multi-scale block complementary mask to detect and learn information of images at different scales.
3. We introduce a pruning and merging network (ToMe) [28] into the ViT architecture, which enhances the throughput of the existing ViT model without the need for training.

## 2. Proposed Method

**Figure 2:**
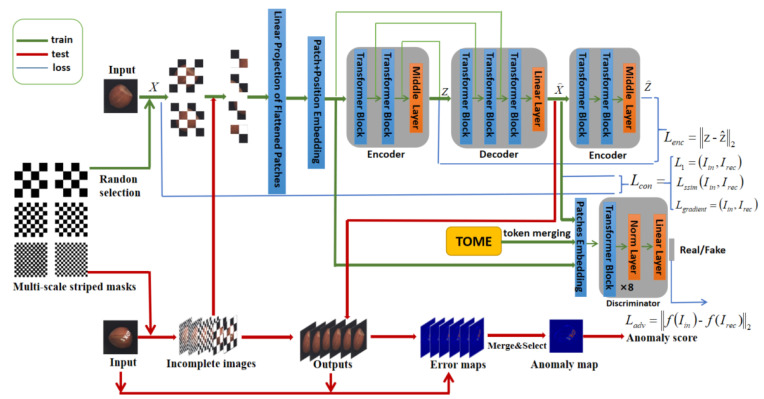
The overall architecture of generative adversarial networks based on U-ViT multi-scale mask feature fusion with pruning and merging. This network uses multi-scale block masks to mask some areas of the input image and reconstructs the image in an adversarial manner. The network is simplified by pruning and merging, and anomaly scores are calculated using error maps.

In this section, we will introduce the proposed framework for metal surface defect detection (as shown in Figure 2). We draw inspiration from generative adversarial models and propose a generative adversarial network with skip connections using the Vision Transformer. Building upon the bar-shaped masks from the SCADN network, we improve upon the design of multi-scale block masks to make them simpler. We introduce pruning and merging to accelerate the network and define multi-scale training and testing strategies, as well as anomaly scoring strategies. During the training process (indicated by the green line in the figure), we randomly select two complementary masks of a certain scale to occlude specific regions of the image for image reconstruction. An additional encoder is added to constrain the latent space features. A discriminator is employed to distinguish between real and generated data. The ToMe network performs pruning and merging on each layer block of the ViT model. During testing (indicated by the red line), we generate multiple masked images by applying three scales of masks to the original image. These masked images are then input to the trained model, resulting in multiple reconstructed images. Each reconstructed image is compared with the original image to generate multiple error maps. Anomaly scores are computed based on the error maps for anomaly detection and localization.

### 2.1. Generative Adversarial Network with a Skip-Connected ViT

#### 2.1.1. Generator Network

As shown in Figure 2, our proposed generative adversarial network is based on GANomaly, where the generator learns the representation of the input data and reconstructs the input data through an encoding and decoding network. An encoding network is added to explicitly learn to minimize the feature distance. The discriminator network D is used to determine whether the input *x* and the output $\hat{x}$ of the generator are real or fake.

**Figure 3:**
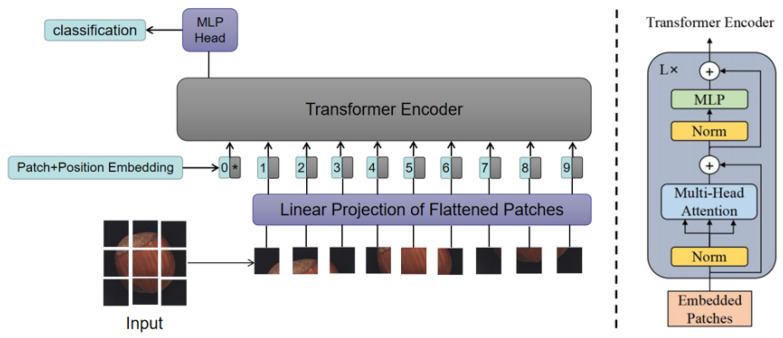
Structure of the ViT model. ViT divides the input image into multiple patches (16 × 16), and then projects each patch into a fixed-length vector and sends it to the Transformer. A special token is added to the input sequence, and the output corresponding to this token is the final category prediction.

Due to the good performance of the ViT in visual tasks, we designed our encoder and decoder using the ViT model, whose structure is shown in Figure 3. The model consists of an embedding layer, a Transformer Encoder, and MLP Head. First, the input image is divided into multiple patches, which are transformed into one-dimensional vectors through linear mapping and input into the embedding layer. After adding position encoding, they are input into the encoder. Layer Norm in the Transformer Encoder standardizes each token, and the multi-head attention mechanism divides the input data into multiple parts, allowing the model to capture different features and patterns in the input data in parallel. MLP improves the model’s ability to fit nonlinear data. The final classification result is obtained through the MLP Head. The decoder also uses the same structure to reconstruct the image.

#### 2.1.2. ViT-Based Cross-Layer Feature Fusion

**Figure 4:**
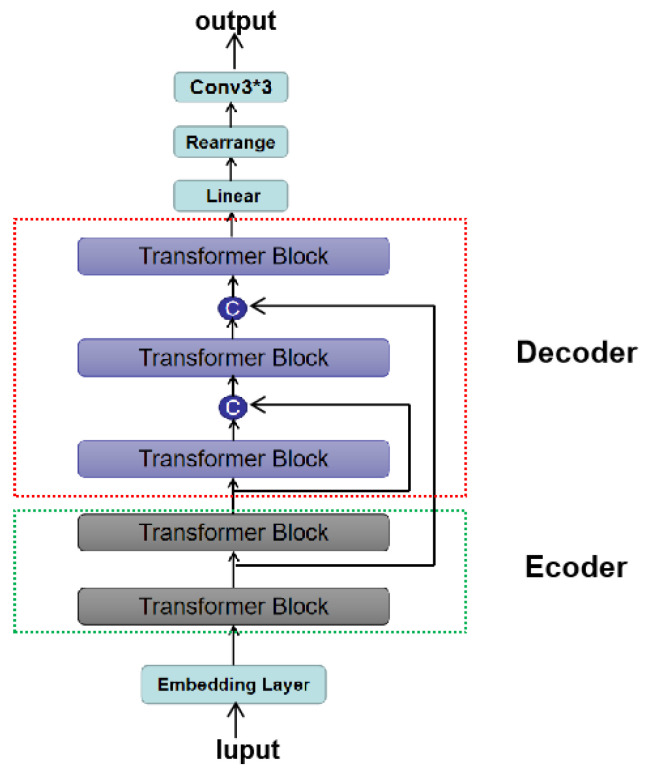
U-ViT architecture with long skip connections between the encoding and decoding layers.

Influenced by the U-Net model structure of CNN networks, we use similar long skip connections between the encoding and decoding layers. Long skip connections can solve the problems of gradient explosion and gradient disappearance that occur during training in deeper networks. They map shallow features to deep layers, allowing information to be flexibly propagated backward and passing image details from shallow layers to top layers for better reconstruction. In our network model, the output of the first Transformer Block of the encoder is connected to the input of the last Transformer Block of the decoder, as shown in Figure 4. Let $h_{m}$, $h_{s}$ be the embeddings from the main branch and the long skip branch, respectively. Before sending them to the next transformer block, they are concatenated and linearly projected as shown in the figure $\left(cocat\left(h_{m},h_{s}\right)\right)$. Additionally, a 3×3 convolutional block is added after the output of the decoder to reduce potential artifacts in the generated images caused by the ViT.

#### 2.1.3. Discriminator Network

The discriminator also uses the ViT model, which consists of a block embedding layer, a Transformer Block, a normalization layer, and a linear mapping layer. Unlike the discriminator in a convolutional neural network, the ViT-based discriminator has a multi-head attention mechanism, and its output is a scalar value that is limited between 0 and 1 after passing through the sigmoid activation function. This makes the discriminator more accurate when judging real and fake values. Through the game between the generator model and the discriminator model, a Nash equilibrium is finally achieved.

### 2.2. Multi-Scale Mask Module

**Figure 5:**
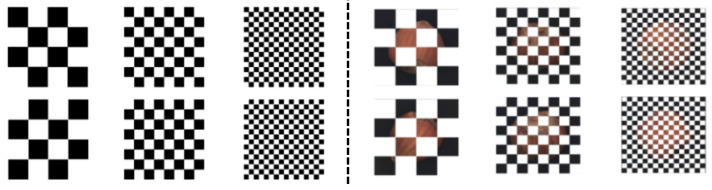
Visualization of the multi-scale block masks.

We designed three different scales of masks, as shown in Figure 5. By using masks to remove certain areas of the image, the network can reconstruct the masked image into a complete normal image. During network training, we need to consider the rich contextual semantic information and the learning time of the network. Therefore, we designed a multi-scale complementary block mask module with six mask images. Unlike the 12 bar masks used in the SCADN, our mask module preserves the total image information while reducing the training time. The multi-scale masking operation can encompass various sizes of anomalies, and the complementary design ensures that semantic information is not lost.

In the multi-scale masking operation, we set the pixel values of the masked regions to 0 and use the white color to indicate the areas to be removed. The ratio of white to black areas is 1:1, and the black and white areas are exchanged to obtain complementary masks, so that each region of the image has an equal chance of being reconstructed. During training, we use a set of two masks of different scales to mask an image twice and feed it into the network. As training progresses, all information in the image is learned. During testing, we use six masks of three scales on each image and feed them into the trained model. Finally, the error score is obtained by merging the error maps.

### 2.3. ToMe Pruning and Merging

Vision Transformer can recognize objects and details in images, but its network is more complex compared to other models such as CNNs, which means it has a slower training speed and requires more computational resources. Therefore, network acceleration models have emerged, and common methods for accelerating Vision Transformer models involve pruning tokens, for example, Efficient Vision Transformers with Dynamic Token Sparsification (DynamicViT) [29], Adaptive Vision Transformers for Efficient Image Recognition (AdaViT) [30], Adaptive Tokens for Efficient Vision Transformer (A-ViT) [31], Enabling Faster Vision Transformers via Latency-Aware Soft Token Pruning (SPViT) [32], and others. Although these methods have good accuracy results, token pruning also has some drawbacks, such as requiring additional training processes and limiting the number of tokens that can be pruned due to the information loss caused by pruning.

**Figure 6:**
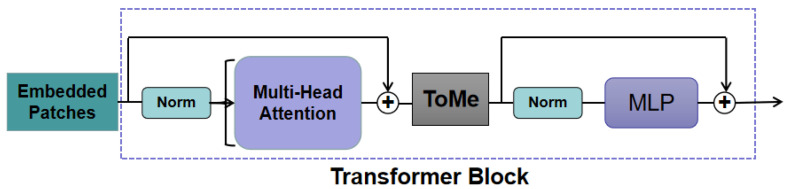
Schematic diagram of the Token Merging model.

The token merging method merges image patches without the need for additional training, striking a balance between performance and speed. The network diagram is shown in Figure 6. The ToMe module operates between the Attention module and the MLP module, using the features in the Attention module to determine which tokens to merge. As the key in the Attention module has summarized the information contained in each token, it is used to measure similarity and determine which tokens to merge, achieving the best trade-off between accuracy and speed.

In this paper, the ToMe network is used as the ViT model in the discriminator, and each layer block is pruned and merged. This results in reducing ‘r’ tokens at each layer. Assuming a Transformer model with ‘L’ layers, merging can lead to a reduction of ‘rL’ tokens. The value of the variable ‘r’ determines the trade-off between speed and accuracy, where fewer tokens imply a lower accuracy but a higher throughput. Similarity is measured using the key from the Attention module to determine which tokens to merge. Experimental results help determine the optimal value of ‘r’ for the model’s performance. Additionally, the discriminator is employed to differentiate between genuine and counterfeit samples generated by the generator, similar to binary classification in image analysis. Therefore, restricting the ToMe model’s use to the classification problem in the discriminator network allows for accelerated training without compromising the image reconstruction quality and network accuracy.

### 2.4. Anomaly Scoring Strategy

#### 2.4.1. Multi-Scale Training Strategy

During the training phase, the encoder maps the image to the latent space. The extracted latent feature vectors are reconstructed into fake images through the decoder. The discriminator distinguishes between the input image and the fake image.

The detailed training process of the network is as follows:

(1) Initialize the weight parameters of the generator and discriminator networks. Firstly, fix the weight of the generator network and update the discriminator.
(2) After the discriminator parameters are updated, keep the discriminator weight unchanged and update the weight parameters of the generator network.

During the parameter update process of the generative adversarial network, we introduce an additional encoder loss $L_{enc}$ to minimize the distance between the input bottleneck features and the encoding features of the generated image. The discriminator uses the Euclidean distance between the intermediate layer feature representations of the input image and the reconstructed image as the adversarial loss $L_{adv}$, reducing the training instability through feature matching. In addition, to enhance the preservation of pixel and detail information in the input image, we introduce pixel consistency loss, structural consistency loss, and gradient consistency loss. Pixel consistency measures the difference in pixels between the input and reconstructed images to improve the image reconstruction ability. Structural consistency uses structural similarity measurements to comprehensively compare the input and reconstructed images in terms of brightness, contrast, and structure. Gradient consistency reflects the frequency of changes in the image, applying gradient constraints to improve the reconstruction quality of the high-frequency components in the image. The consistency loss defined by the combination of the three is as follows:

(1) $$L_{con}=L_{1}\left(I_{in},I_{rec}\right)+L_{ssim}\left(I_{in},I_{rec}\right)+L_{gradient}\left(I_{in},I_{rec}\right)$$

(2) $$L_{1}\left(I_{in},I_{rec}\right)={∥I_{in}-I_{rec}∥}_{1}$$

(3) $$L_{ssim}\left(I_{in},I_{rec}\right)=1-SSIM\left(I_{in},I_{rec}\right)$$

(4) $$L_{gradient}\left(I_{in},I_{rec}\right)={∥\left(▽I_{in},▽I_{rec}\right)∥}_{1}$$

where $I_{in}$ represents the input image, $I_{rec}$ represents the reconstructed image, and ∥··∥ represents the $L_{1}$ norm. SSIM represents the structural similarity measurement function; the larger the value of SSIM, the higher the similarity between the two images. ▽ represents the image gradient operation.

Combining the encoder loss, adversarial loss, and contextual loss, we obtain the total loss for updating the weight parameters of the generative adversarial network. The expression for the total loss is:

(5) $$L_{total}=\alpha_{1}L_{adv}+\alpha_{2}L_{enc}+\alpha_{3}L_{con}$$

where $\alpha_{1}$, $\alpha_{2}$, and $\alpha_{3}$ represent the weight coefficients of the adversarial loss, encoder loss, and consistency loss.

#### 2.4.2. Multi-Scale Testing Strategy

**Figure 7:**
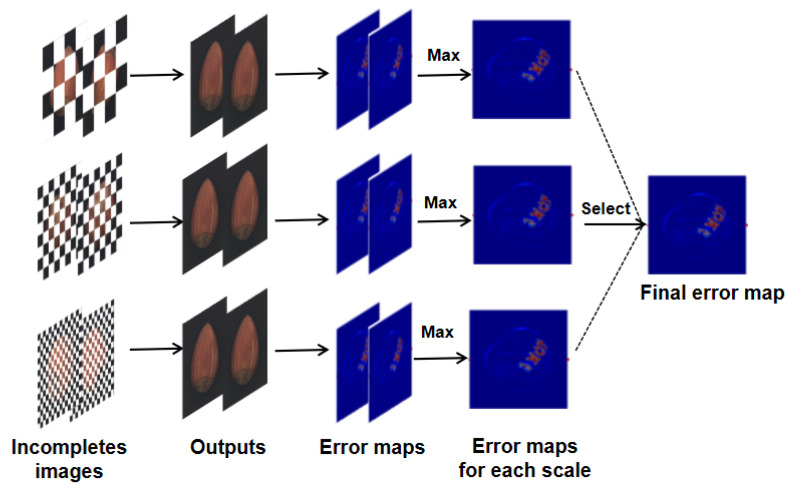
Test strategy diagram.

During testing, we merge multiple error map outputs to calculate the final error map. The merging process of the error map is shown in Figure 7. The large-scale mask can detect large anomalies, while the small-scale mask is more sensitive to small anomalies. By merging the reconstruction results at multiple complementary positions of different scales, the overall performance of anomaly detection can be significantly improved.

First, for the input image *I*, multiple mask images $M_{ij}$ (*i* = 0, 1, 2 represent three block-shaped mask scales. *j* = 1, 2 represent two complementary masks of the same scale) are assigned. The image is input into the generator after being processed by two complementary masks of the same scale, and the reconstructed images $O_{i1}$ and $O_{i2}$ are output. Considering only the reconstructed part of the masked area, the two images are added and merged to obtain a complete reconstruction image.

(6) $$O_{i}=\left(O_{i1}*M_{i1}\right)+\left(O_{i2}*M_{i2}\right)$$

(7) $$E_{i}=\left(O_{i}-I\right)$$

where $E_{i}$ represents the error map generated between the input image of the network and the fused output image.

Three error maps are obtained by merging the output of the three scales. To merge error maps of different scales, we first calculate the average value of the error maps at different scales as a reference value, denoted as $\mu_{i}$. Then, we select the error map with the maximum distance from $\mu_{i}$ as the final error map.

(8) $$f=argmax(average\left(E_{i}\right)-\mu_{i})$$

(9) $$E_{final}=E_{f}$$

where $E_{final}$ represents the final error plot, and the anomaly score can be calculated.

## 3. Experiments

In this section, we conduct experiments on five metal datasets and the MVTec AD [33] dataset using our proposed method. We also discuss some training details and evaluation metrics used in the experiments. Our method is compared with defect detection algorithms such as GANomaly, SCADN, DRAEM, and Patchore.

### 3.1. Dataset and Evaluation Metrics

The five metal datasets used in this experiment include publicly available datasets and collected data, including Kole electronic commutators, rail surfaces, steel, metal parts, and metal shim. There are a total of 2778 high-resolution images, including 1798 training images and 980 testing images, organized in the format of the MVTec AD dataset. The training set only contains normal samples, while the test set contains both normal and abnormal samples.

**Figure 8:**
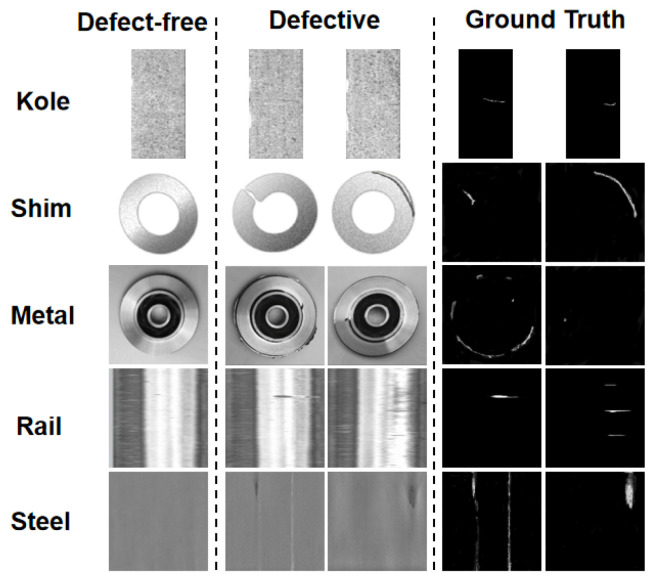
Defect-free and defective samples in the metal dataset.

The MVTec AD dataset is mainly used for unsupervised surface anomaly detection tasks, with a total of 5354 high-resolution images, including 15 categories: 5 different texture categories and 10 different object categories. Each dataset is divided into a training set and a test set, with the training set containing only defect-free images and the test set containing both defect-free and 70 types of defective images. As shown in Figure 8, it includes five metal datasets with both defect-free and defective samples.

For the evaluation metrics of image surface, we used the image-level AUC and the F1-score. The AUC is the area under the receiver operating characteristic (ROC) curve, which has a true positive rate on the horizontal axis and a false positive rate on the vertical axis. The F1-score is the harmonic mean of precision and recall, with a maximum value of 1 and a minimum value of 0.

### 3.2. Implementation Details

The network encoder and decoder we designed consist of 13 Transformer blocks and a linear layer, and the encoder with the constraint of latent features has the same parameters as the encoder in the generator. The discriminator consists of eight Transformer blocks, a middle layer, and a linear layer. For the training stage of the generator and discriminator networks, we set the batch size to 8, the number of network training iterations to 500, and used Adamw [34] as the optimizer with a learning rate of $1\times 10^{-4}$. The weight of the total loss $L_{total}$ was set to $\alpha_{1}=1$, $\alpha_{2}=1$, $\alpha_{3}=20$. All experiments in this paper were conducted using PyTorch 1.8.0, CUDA 11.1, and CUDNN 8.0.5 frameworks. All experiments were trained and tested on a system equipped with an Intel Core i9-10900K CPU, 64 GB RAM, and an NVIDIA GeForce RTX 3090 GPU. The manufacturer of the purchased graphics card is NVIDIA Corporation, headquartered in Santa Clara, CA, USA.

### 3.3. Comparison with Existing Methods

In this section, we compare our proposed method with several state-of-the-art approaches. Table 1 and Table 2 present the AUC [35] and F1-score [36] on five metal datasets. Our method achieves better results in terms of the average values and outperforms other comparison results in multiple categories, demonstrating the effectiveness of our approach.

As shown in the table below, we first compare the performance of our proposed method with several other defect detection methods on the five metal datasets: GANomaly, MAE, Hybrid Convolution-Transformer Model (ConvMAE) [37], SCADN, DRAEM, Patchore, Student-Teacher, Reverse-Distillation, Coupled Superball-based Feature Adaptive Anomaly Localization Network (CFA) [38], Padim, Fully Convolutional Cross-Scale-Flows (Csflow) [39], and Localization via 2D Normalizing Flows (Fastflow) [40].

Our method shows significant advantages on the datasets. Our model achieves 100% accuracy on the Rail and Shim datasets. Particularly on the Shim dataset, compared to the Patchore and Padim models, which have excellent accuracy, our method improves the AUC by 6.5% and 0.1%, and the F1-score by 2.3% and 0.3%, respectively. On the Steel dataset, our AUC results are 1.3% and 0.9% higher than the ConvMAE and Padim models, and the F1-score results are 5.1% and 1.6% higher. Similarly, our model performs exceptionally well on the Metal dataset, achieving an AUC of 0.993 and an F1-score of 0.997, outperforming all the compared models. It surpasses the performance of the Padim model by 1.8% in the AUC and 1.2% in the F1-score. These results demonstrate the superiority of our model. For the Kolektor dataset, our AUC and F1-score results are 0.988 and 0.942, respectively, which are not as high as the Patchore model’s 100% accuracy. This is because the Kolektor dataset includes defects such as microscopic fragments or cracks. Images of very small cracks resemble defect-free images, and our model’s powerful feature extraction capability may lead to misjudgments of such defects. From the average experimental results on the five metal category datasets, our method outperforms the existing best methods with an average improvement of 0.6% in the AUC and 0.3% in the F1-score, achieving the best results. This is attributed to our proposed approach of using the ViT model and multi-scale information learning, which demonstrates a more comprehensive feature extraction capability and provides further supplementation for detailed features. Our model can accurately reconstruct and detect anomalies in the presence of defects such as rust, cracks, wear, and damage to metal surfaces.

**Figure 9:**
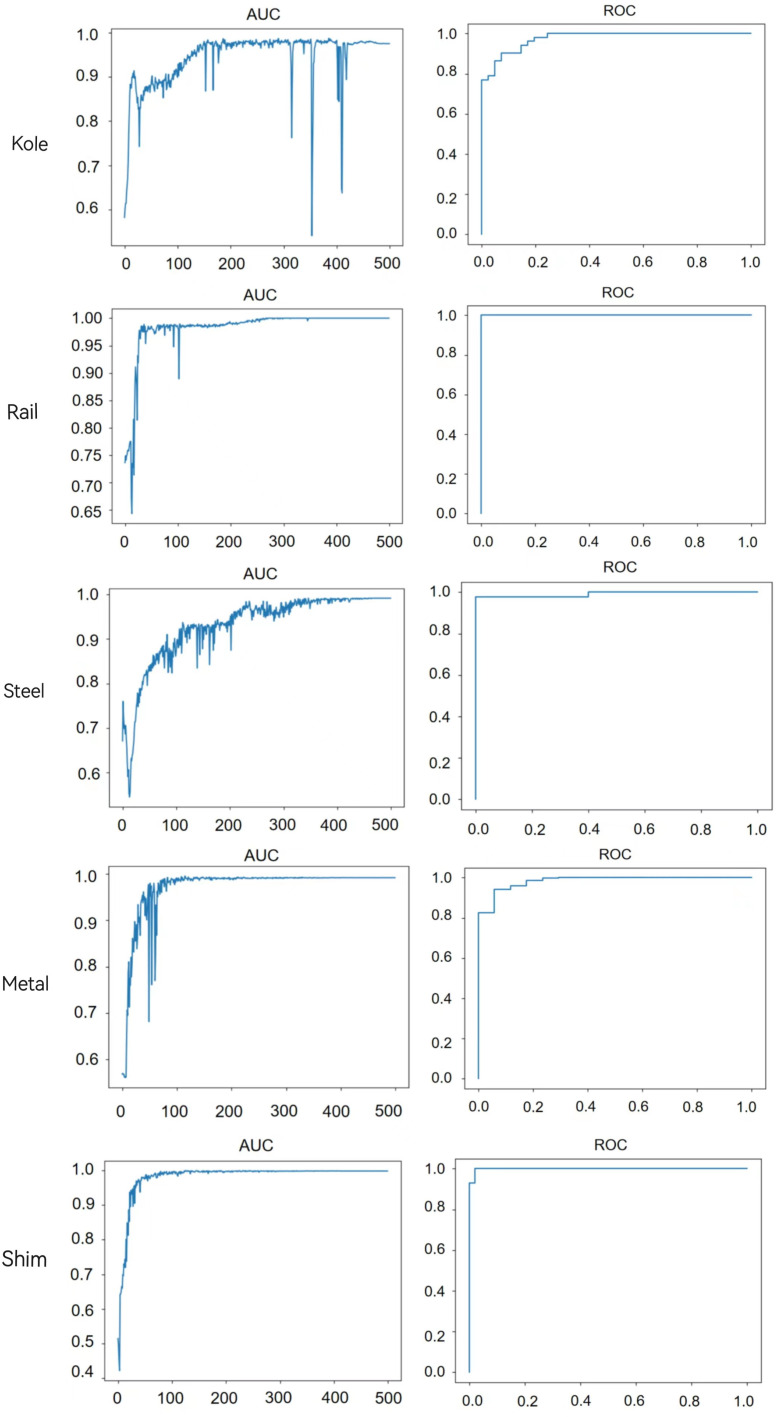
AUC and ROC line chart on the five metal datasets.

To clearly demonstrate the training process and superiority of our method on each dataset, we have plotted the AUC and ROCs for the five metal datasets. Figure 9 provides a visual representation of the training process and results after 500 epochs. It is evident from the figure that the model achieves stable and excellent results after 500 rounds of training and testing on all datasets.

Our method not only demonstrates superiority on the aforementioned metal datasets but also shows strong competitiveness on the MvTec AD dataset. Table 3 and Table 4 display the AUC and F1-score results for different category subsets of the MVTec AD dataset. In terms of image-reconstruction-based methods, we compared our method with those based on convolutional neural network structures and Transformer structures. Our method achieves the best results, with an average AUC of 0.921 and an average F1-score of 0.909. For the categories of bottle, grid, pill, tile, toothbrush, and zipper, our AUC results are 2.4%, 1.7%, 10.5%, 17.9%, 1.9%, and 12.5% higher than SCADN, respectively. The F1-score results are 4.2%, 2%, 1.1%, 2.3%, 0.1%, and 1% higher than SCADN, respectively. For the capsule and leather categories, our AUC and F1-score results are 10.3%, 1.4%, and 18.1%, 6.1% higher than GANomaly, respectively. For the hazelnut category, our AUC and F1-score improve by 1.4% and 1.6%, respectively, compared to the VT-ADL model. However, our model performs poorly on the cable and carpet categories. This is because the cable and carpet datasets have irregular textures, requiring more resources to handle, which poses a challenge for our network.

### 3.4. Result Visualization

**Figure 10:**
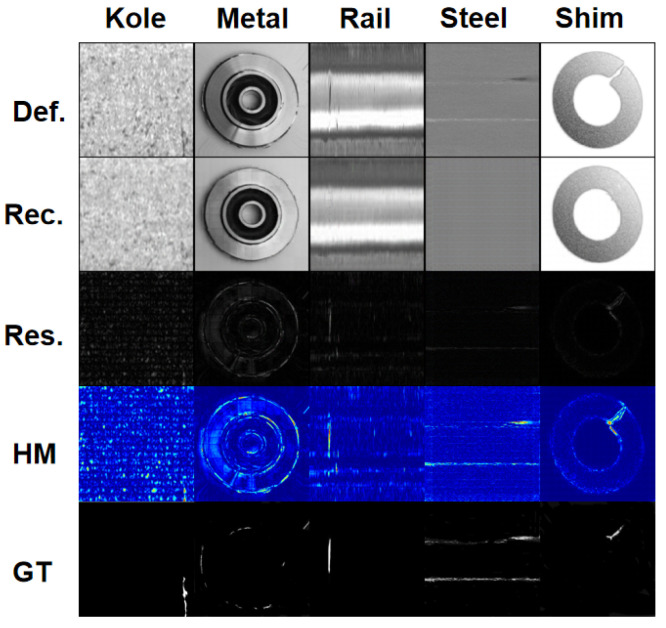
Test results on the five metal datasets. Def. represents the defect image, Rec. represents the reconstructed image, Res. represents the residual image, HM represents the heat map, and GT represents the label of the true defect.

**Figure 11:**
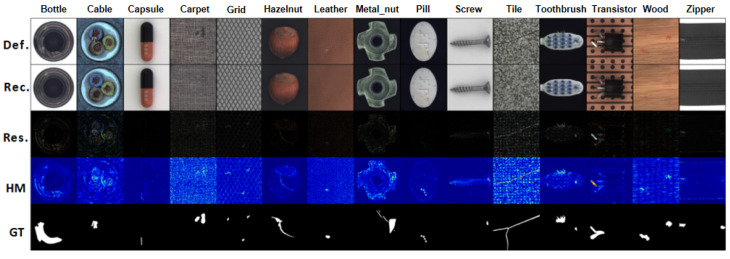
Test results on the MVTec AD dataset. Def. represents the defect image, Rec. represents the reconstructed image, Res. represents the residual image, HM represents the heat map, and GT represents the label of the true defect.

To compare the reconstruction results, we visualize the output images, error maps, and heatmaps generated by our method in Figure 10 and Figure 11. From the results, it is evident that our method can perfectly reconstruct defective images into defect-free images. By utilizing the residual mapping between the defective image and the reconstructed image, we can identify the defects. By comparing the heatmaps with the labels, our model can accurately detect the locations of the defects, and our output images bear a closer resemblance to the normal samples. This demonstrates that our method can efficiently explore image features from normal patterns and focus on anomalous locations. For various types of defects present in Kolektor, such as cracks, missing areas in metal, wear in rails, rust in steel, deformation in shim, as well as the diverse range of defect types in the MVTec AD dataset, our network can perfectly reconstruct and accurately locate anomalies.

### 3.5. The Effect of Pruning and Merging Tokens

This section primarily discusses the impact of the quantity of pruning and merging on the model. In the token merging network used in the discriminator, we chose to merge tokens instead of pruning them. In the Transformer model, merging is performed to reduce R tokens in each layer block. The value of the variable R determines the trade-off between speed and accuracy, as fewer tokens result in a lower accuracy but a higher throughput. Therefore, we conducted experiments to determine the appropriate value of R. Table 5 shows the variations in the AUC and F1-score when setting different values of R, namely 0, 1, 2, 4, and 8. Within a certain range, as the value of R increases, the accuracy of the results remains relatively stable. However, when R exceeds 4, there is a slight decrease in the AUC and F1-score. Therefore, an appropriate amount of pruning and merging can improve the training speed while maintaining accuracy. Based on comprehensive experiments, when R is set to 2, reducing 2 tokens per layer block, the network can be accelerated without impacting the accuracy of the results.

### 3.6. The Impact of Weight Coefficients of the Loss Functions

In this section, we discuss the impact of weight coefficients for consistency losses on the model. To enhance the preservation of pixel and detail information in the input images, we introduce the pixel consistency loss, structural consistency loss, and gradient consistency loss. Together, they form the context loss $L_{con}$, where $\alpha_{1}$, $\alpha_{2}$, and $\alpha_{3}$ represent the weight coefficients for the adversarial loss, encoder loss, and context loss in the network, respectively. A higher weight for the context loss can generate images that are closer to the original images. A higher weight for the encoder loss can enhance the semantic consistency, and a higher weight for the adversarial loss can produce more realistic images. However, excessively high weights may lead to overfitting or unstable training. Therefore, the choice of weight coefficients depends on the specific task and dataset. Based on previous experience, we conducted experiments and performed iterations to optimize the best combination of weight coefficients. As shown in Table 6, we initially set all weight coefficients to 1. Additionally, since we prioritized the training of the context loss function to ensure that the generated images are closer to the original images, we kept $\alpha_{1}$ and $\alpha_{2}$ unchanged and adjusted the weight coefficient of the context loss, denoted as $\alpha_{3}$. We conducted experiments with four modes of C, i.e., 1, 10, 20, and 30, to verify the results. The findings indicate that when the pruning and merging quantity is maintained at R = 2, our network achieves the optimal AUC and F1-score results when $\alpha_{3}$ = 20.

### 3.7. Ablation Study

In this section, a series of ablation experiments were conducted on a dataset of five metal types to demonstrate the effectiveness of the different modules in the proposed surface defect detection method. We primarily validated the effectiveness of individual strategies in the proposed model from four aspects: the effectiveness of multi-scale masking, the effectiveness of the loss function module, the effectiveness of the consistency loss, and the effectiveness of pruning and merging.

#### 3.7.1. The Effectiveness of Multi-Scale Masks

To validate the effectiveness of the multi-scale masking module, a generative adversarial network based on a Transformer with cross-layer feature fusion was used. Three complementary block masks of different scales (0, 1, 2) were designed. Experiments were conducted with five combinations of masks, 0, 1, 2, 0/1, and 0/1/2, as shown in Table 7.

Based on the results in Table 7, it can be observed that the use of different mask scales indeed has a significant impact on surface defect detection. The dataset includes five different categories and various defect types, covering small or large portions of the images. Therefore, considering defects of different sizes, the mixed masking approach using 0, 1, and 2 can provide a more accurate and representative evaluation of the defects, leading to an optimal performance in defect detection.

#### 3.7.2. The Effectiveness of Loss Function Module

Our network achieves Nash equilibrium in the generation and adversarial processes. Without changing the combination of masks, we considered three aspects of the loss functions in our network. The context loss function and encoder loss function help maintain the similarity and consistency between the generated images and the original images, while the adversarial loss function drives the generator to produce realistic images. These three loss functions work together to enable the generator to generate realistic images that are similar to real images. The U-net utilizes mechanisms such as feature extraction, upsampling, skip connections, and multi-scale processing to propagate image details from shallow layers to top layers, enabling high-quality extraction of image information. In order to validate the effectiveness of each loss module, we conducted relevant ablation experiments, and the results are shown in Table 8.

From the experimental results, it can be concluded that each loss plays an important role in the network. Under the joint constraint of the three loss functions, the network produces optimal reconstructed images as well as the best AUC and F1-score results. Additionally, it can be observed that the inclusion of skip connection modules further improves the accuracy of the results.

#### 3.7.3. The Effectiveness of Consistency Loss

Without changing other network structures, in the context loss, we combined pixel consistency, structural consistency, and gradient consistency. In the generator network, we used the pixel consistency loss function $L_{1}$ and evaluated its impact on the image reconstruction ability. We added the structural consistency loss function $L_{ssim}$ as the generator loss function to evaluate the generation effect $L_{1}+L_{ssim}$. By combining pixel consistency, structural consistency, and gradient consistency, we formed the consistency loss function $L_{1}+L_{ssim}+L_{gradient}$ to evaluate the reconstruction ability and generation effect in the context loss. The results are shown in Table 9.

On the five categories of metal datasets, using a single loss function and combining three loss functions with consistency loss achieved the best performance. This effectively constrains the image in terms of pixels, structure, and gradients.

#### 3.7.4. The Effectiveness of Pruning and Merging

To validate the effectiveness of pruning and merging, we use the most intuitive metric, the training time, to represent the performance after pruning. We compared the performance without pruning and merging (R = 0) with the case where two tokens are removed from each layer block (R = 2). With the other parameters unchanged, the results are shown in Table 10.

On the dataset of five metal types, pruning and merging reduces the average training time by 10%. With a batch size of 8, the maximum amount of data processed per training iteration is reduced by an average of 8%. Additionally, a comparison experiment with the SCADN validated the significant advantage of simpler, block-wise complementary masks over excessive stripe masks in terms of the training time.

## 4. Conclusions

We studied the problem of surface defect detection in metal industrial products, which have various types of surface defects. Problems in defect detection include difficulties in collecting defect samples, weak network feature extraction abilities, and low training accuracies. We propose a semi-supervised anomaly detection method based on image reconstruction with a pruned–merged generative adversarial network using Transformer-based multi-scale masked feature fusion to solve this problem. We design six complementary multi-scale block masks to remove certain areas from normal samples, and incorporate long skip connections in the generator structure to obtain more information and reconstruct missing regions in an adversarial manner to match the input image. In testing, we use an error map to infer anomalous samples, calculated as the difference between the reconstructed images of normal and anomalous samples and the input image. By using pruning and merging on the blocks of the ViT, we can speed up network training and achieve faster training speeds. Additionally, we use a consistency loss to constrain the network by utilizing the differences in pixels, structure, and gradients between defect images and reconstructed images, further improving the performance of defect detection. We conducted various comprehensive experiments and achieved state-of-the-art performance compared to existing methods, with an average AUC of 0.995 and an F1-score of 0.986 on five categories of metal datasets. When applied to the MVTec AD dataset, our method also achieved good results, outperforming most reconstruction-based methods, notably with a 100% accuracy in the AUC and F1-score for the screw category. This proves the effectiveness of our method.

This method currently focuses on detecting defects in metal industrial products, and in future research, we will apply it to surface defect detection in other industrial products. We will continue to optimize and simplify the network to improve the training accuracy and speed. We will also explore more practical applications of the network in production and daily life.

## Figures and Tables

**Table 1:** Comparison of different methods regarding the AUC on five metal datasets. (1: GANomaly, 2: MAE, 3: ConvMAE, 4: SCADN, 5: DRAEM, 6: Patchore, 7: Student-Teacher, 8: Reverse-Distillation, 9: CFA, 10: Padim, 11: Csflow, 12: Fastflow). Bold numbers represent the optimal results.

CAT	1	2	3	4	5	6	7	8	9	10	11	12	Ours
Kole	0.752	0.768	0.618	0.809	0.813	**1.000**	0.833	0.726	0.644	0.985	0.816	0.697	0.988
Rail	0.581	0.901	0.582	0.764	0.966	**1.000**	0.986	0.843	0.998	**1.000**	0.984	0.548	**1.000**
Steel	0.953	0.850	0.983	0.907	0.941	0.998	0.893	0.719	0.928	0.987	**0.999**	0.593	0.996
Metal	0.855	0.791	0.879	0.993	0.856	0.906	0.976	0.468	0.924	0.975	0.971	0.960	**0.993**
Shim	0.869	0.745	0.748	0.957	0.892	0.935	0.857	0.606	0.988	0.999	0.981	0.996	**1.000**
Avg	0.802	0.811	0.762	0.886	0.894	0.968	0.909	0.672	0.896	0.989	0.950	0.759	**0.995**

**Table 2:** Comparison of different methods regarding the F1-Score on five metal datasets. (1: GANomaly, 2: MAE, 3: ConvMAE, 4: SCADN, 5: DRAEM, 6: Patchore, 7: Student-Teacher, 8: Reverse-Distillation, 9: CFA, 10: Padim, 11: Csflow, 12: Fastflow). Bold numbers represent the optimal results.

CAT	1	2	3	4	5	6	7	8	9	10	11	12	Ours
Kole	0.717	0.752	0.722	0.904	0.783	**1.000**	0.834	0.834	0.967	0.954	0.788	0.736	0.942
Rail	0.955	0.959	0.955	0.965	0.977	**1.000**	0.988	0.966	0.979	**1.000**	0.977	0.941	**1.000**
Steel	0.972	0.947	0.947	0.967	0.989	0.981	0.978	0.838	0.942	0.982	**0.998**	0.947	**0.998**
Metal	0.981	0.982	0.982	0.994	0.983	0.958	0.992	0.981	0.875	0.985	0.991	0.980	**0.997**
Shim	0.765	0.683	0.667	0.889	0.876	0.972	0.786	0.669	0.444	0.992	0.905	0.984	**0.995**
Avg	0.878	0.865	0.855	0.944	0.922	0.982	0.916	0.858	0.841	0.983	0.932	0.918	**0.986**

**Table 3:** Comparison of different methods regarding the AUC on the MVTec AD dataset. Bold numbers represent the optimal results.

CAT	GANomaly	Skip-Ganomaly	MAE	ConvMAE	VT-ADL	SCADN	Ours
bottle	0.791	0.811	0.790	0.884	0.949	0.957	**0.981**
cable	0.775	0.837	0.679	0.599	0.776	**0.856**	0.843
capsule	0.774	0.768	0.629	0.663	0.672	0.765	**0.877**
carpet	0.822	**0.899**	0.640	0.215	0.370	0.504	0.781
grid	0.871	0.966	0.916	0.958	0.871	0.983	**1.000**
hazelnut	0.775	0.791	0.830	0.641	0.897	0.833	**0.911**
leather	0.804	0.786	0.685	0.364	0.728	0.659	**0.985**
metal-nut	0.572	0.731	0.761	**0.857**	0.726	0.624	0.824
pill	0.747	0.689	0.675	0.703	0.705	0.814	**0.919**
screw	**1.000**	0.998	0.257	**1.000**	0.900	0.800	**1.000**
tile	0.723	0.733	0.720	0.766	0.796	0.792	**0.971**
toothbrush	0.704	0.742	0.740	0.522	0.901	0.981	**1.000**
transistor	0.833	0.785	0.683	**0.896**	0.796	0.863	0.895
wood	0.921	0.937	0.653	0.904	0.781	**0.968**	0.961
zipper	0.744	0.657	0.725	0.710	0.808	0.846	**0.971**
Avg	0.790	0.809	0.692	0.712	0.807	0.818	**0.921**

**Table 4:** Comparison of different methods regarding the F1-score on the MVTec AD dataset. Bold numbers represent the optimal results.

CAT	GANomaly	Skip-Ganomaly	MAE	ConvMAE	VT-ADL	SCADN	Ours
bottle	0.863	0.863	0.863	0.863	0.932	0.913	**0.955**
cable	0.777	0.789	0.765	0.763	0.749	0.814	**0.843**
capsule	0.904	0.905	0.905	0.924	0.871	0.911	**0.928**
carpet	0.864	0.864	0.864	0.864	0.834	**0.865**	**0.865**
grid	0.859	0.844	0.885	0.844	0.916	0.974	**0.992**
hazelnut	0.782	0.805	0.778	0.778	0.857	0.822	**0.873**
leather	0.852	0.853	0.852	0.852	0.877	0.852	**0.913**
metal-nut	0.894	0.901	0.895	0.894	0.902	0.894	**0.915**
pill	0.915	0.919	0.915	0.915	0.898	0.927	**0.950**
screw	0.873	0.958	0.853	0.992	0.911	0.921	**1.000**
tile	0.836	0.836	0.836	0.841	0.849	0.841	**0.864**
toothbrush	0.831	0.833	0.833	0.833	0.891	0.895	**0.896**
transistor	0.593	0.697	0.624	0.756	0.801	0.719	**0.822**
wood	0.863	0.918	0.899	0.894	0.893	0.922	**0.937**
zipper	0.881	0.882	0.888	0.885	0.903	0.909	**0.919**
Avg	0.839	0.858	0.844	0.860	0.872	0.879	**0.911**

**Table 5:** Comparison of AUC and F1-score with different values of R on the metal datasets. Bold numbers represent the optimal results.

CAT	R = 0		R = 1		R = 2		R = 4		R = 8	
	AUC	F1-Score	AUC	F1-Score	AUC	F1-Score	AUC	F1-Score	AUC	F1-Score
Kole	0.981	0.932	0.979	0.922	0.984	0.934	0.965	0.919	0.969	0.919
Rail	0.993	0.989	0.993	0.989	0.993	0.989	0.993	0.981	0.993	0.969
Steel	0.994	0.998	0.994	0.998	0.994	0.998	0.985	0.978	0.965	0.978
Metal	0.991	0.995	0.989	0.994	0.991	0.995	0.992	0.995	0.991	0.995
Shim	1.000	0.991	1.000	0.991	1.000	0.992	0.999	0.971	0.999	0.971
Avg	**0.992**	0.981	0.991	0.979	**0.992**	**0.982**	0.987	0.969	0.983	0.966

**Table 6:** Comparison of the AUC and F1-score with different weight coefficients on the metal datasets. Bold numbers represent the optimal results.

CAT	$\alpha_{1}$ = 1, $\alpha_{2}$ = 1, $\alpha_{3}$ = 1		$\alpha_{1}$ = 1, $\alpha_{2}$ = 1, $\alpha_{3}$ = 10		$\alpha_{1}$ = 1, $\alpha_{2}$ = 1, $\alpha_{3}$ = 20		$\alpha_{1}$ = 1, $\alpha_{2}$ = 1, $\alpha_{3}$ = 30	
	AUC	F1-Score	AUC	F1-Score	AUC	F1-Score	AUC	F1-Score
Kole	0.961	0.913	0.969	0.919	0.988	0.942	0.988	0.942
Rail	0.995	0.987	0.994	0.985	1.000	1.000	0.999	0.992
Steel	0.955	0.979	0.948	0.972	0.996	0.998	0.996	0.998
Metal	0.937	0.985	0.951	0.992	0.993	0.997	0.935	0.991
Shim	0.999	0.988	1.000	0.985	1.000	0.995	0.999	0.975
Avg	0.969	0.970	0.972	0.971	**0.995**	**0.986**	0.983	0.980

**Table 7:** Comparison of the AUC and F1-score under different mask settings on the metal datasets. Bold numbers represent the optimal results.

CAT	Mask = 0		Mask = 1		Mask = 2		Mask = 0/1		Mask = 0/1/2	
	AUC	F1-Score	AUC	F1-Score	AUC	F1-Score	AUC	F1-Score	AUC	F1-Score
Kole	0.977	0.897	0.969	0.962	0.938	0.926	0.981	0.927	0.988	0.942
Rail	0.992	0.985	1.000	0.996	1.000	0.988	0.993	0.981	1.000	1.000
Steel	0.986	0.978	0.983	0.977	0.972	0.972	0.994	0.989	0.996	0.998
Metal	0.990	0.994	0.965	0.993	0.950	0.992	0.989	0.991	0.993	0.997
Shim	0.966	0.945	1.000	0.989	0.999	0.979	1.000	0.985	1.000	0.995
Avg	0.982	0.960	0.983	0.983	0.972	0.971	0.991	0.975	**0.995**	**0.986**

**Table 8:** Ablation study results of the loss function module on the metal datasets. Bold numbers represent the optimal results.

CAT	$L_{\mathrm{con}}$		$L_{\mathrm{con}}+L_{\mathrm{adv}}$		$L_{\mathrm{con}}+L_{\mathrm{adv}}+L_{\mathrm{enc}}$		$L_{\mathrm{con}}+L_{\mathrm{adv}}+L_{\mathrm{enc}}+\mathrm{Unet}$	
	AUC	F1-Score	AUC	F1-Score	AUC	F1-Score	AUC	F1-Score
Kole	0.978	0.919	0.972	0.919	0.987	0.942	0.988	0.942
Rail	0.985	0.981	0.995	0.984	0.991	0.992	1.000	1.000
Steel	0.993	0.988	0.995	0.989	0.996	0.998	0.996	0.998
Metal	0.956	0.991	0.991	0.995	0.993	0.997	0.993	0.997
Shim	1.000	0.995	1.000	0.986	1.000	0.994	1.000	0.995
Avg	0.982	0.975	0.991	0.975	0.993	0.985	**0.995**	**0.986**

**Table 9:** Ablation study results of the consistency loss on the metal datasets. Bold numbers represent the optimal results.

CAT	$L_{1}$		$L_{1}+L_{\mathrm{ssim}}$		$L_{1}+L_{\mathrm{ssim}}+L_{\mathrm{gradient}}$	
	AUC	F1-Score	AUC	F1-Score	AUC	F1-Score
Kole	0.986	0.937	0.977	0.913	0.988	0.942
Rail	0.992	0.969	0.992	0.973	1.000	1.000
Steel	0.969	0.978	0.981	0.989	0.996	0.998
Metal	0.969	0.991	0.972	0.992	0.993	0.997
Shim	1.000	0.999	1.000	0.995	1.000	0.995
Avg	0.983	0.975	0.988	0.972	**0.995**	**0.986**

**Table 10:** Comparison of training time and data volume with and without pruning and merging on the metal datasets.

CAT		R=0	R=1	SCADN
Kole	Train Time	4 h 53 min	4 h 22 min	6 h 17 min
Rail	Train Time	4 h 25 min	3 h 59 min	5 h 03 min
Steel	Train Time	4 h 42 min	4 h 13 min	5 h 58 min
Metal	Train Time	4 h 39 min	4 h 14 min	6 h 01 min
Shim	Train Time	11 h 31 min	10 h 27 min	14 h 24 min

## Data Availability

This study analyzed the koletor sdd dataset, RSDDs rail surface defect dataset, steel-belt dataset, metal cast dataset, metal shim dataset, and MVTec AD anomaly detection public dataset.
